# Characterization of Synovial Cytokine Patterns in Bucket-Handle and Posterior Horn Meniscal Tears

**DOI:** 10.1155/2020/5071934

**Published:** 2020-10-22

**Authors:** Marco Turati, Davide Maggioni, Nicolò Zanchi, Marta Gandolla, Massimo Gorla, Paola Sacerdote, Silvia Franchi, Laura Rizzi, Alessandra Pedrocchi, Robert J. Omeljaniuk, Giovanni Zatti, Antonio Torsello, Marco Bigoni

**Affiliations:** ^1^Orthopedic Department, San Gerardo Hospital, Monza, Italy; ^2^School of Medicine and Surgery, University of Milano-Bicocca, Monza, Italy; ^3^Transalpine Center of Pediatric Sports Medicine and Surgery, University of Milano-Bicocca, Monza, Italy, Hospital Couple Enfant, Grenoble, France; ^4^Department of Paediatric Orthopedic Surgery, Hopital Couple Enfants, Grenoble Alpes University, Grenoble, France; ^5^Politecnico di Milano, NearLab, Department of Electronics, Information and Bioengineering, Milan, Italy; ^6^Department of Pharmacological and Biomolecular Sciences, University of Milan, Milan, Italy; ^7^Department of Biology, Lakehead University, Thunder Bay, ON, Canada P7B5E1

## Abstract

The specific etiology of meniscal tears, including the mechanism of lesion, location, and orientation, is considered for its contribution to subsequent joint cytokine responsiveness, healing outcomes, and by extension, appropriate lesion-specific surgical remediation. Meniscal repair is desirable to reduce the probability of development of posttraumatic osteoarthritis (PTOA) which is strongly influenced by the coordinate generation of pro- and anti-inflammatory cytokines by the injured cartilage. We now present biochemical data on variation in cytokine levels arising from two particular meniscal tears: bucket-handle (BH) and posterior horn (PH) isolated meniscal tears. We selected these two groups due to the different clinical presentations. We measured the concentrations of TNF-*α*, IL-1*β*, IL-6, IL-8, and IL-10 in knee synovial fluid of 45 patients with isolated meniscal lesions (BH tear, *n* = 12; PH tear, *n* = 33). TNF-*α* levels were significantly (*p* < 0.05) greater in the BH group compared with the PH group, whereas IL-1*β* levels were significantly greater (*p* < 0.05) in the PH group compared with the BH group. Both BH and PH groups were consistent in presenting a positive correlation between concentrations of IL-6 and IL-1*β*. A fundamental difference in IL-10 responsiveness between the two groups was noted; specifically, levels of IL-10 were positively correlated with IL-6 in the BH group, whereas in the PH group, levels of IL-10 were positively correlated with IL-1*β*. Collectively, our data suggest a possible influence of the meniscal tear pattern to the articular cytokine responsiveness. This differential expression of inflammatory cytokines may influence the risk of developing PTOA in the long term.

## 1. Introduction

Meniscal damage is associated with a 6-fold increased risk of developing osteoarthritis (OA) diagnosed by standard radiographic imaging [1]. The incidence of posttraumatic osteoarthritis (PTOA) is greater in young to middle-aged adults compared to the elderly due to the former's greater sporting activity [2]. Among the traumatic lesions of the knee, meniscal injuries are the most common source of pain and disability [3, 4] and also increase by 50% the probability of developing radiographic signs of PTOA within 10-20 years after meniscectomy [5].

Meniscal injuries lead to mechanical alterations of the affected joint which are typically remediated by surgical intervention [6, 7]. Meniscal preservation has been suggested as the treatment of choice since it has been known for a long time that partial meniscectomy is strongly correlated with the development of early OA in children [8]. In addition, recent studies indicate that increased synovial fluid inflammatory cytokines, consequent to meniscal tears, may alter interactions between different articular tissues [9] and may lead to development of PTOA [10–12]. The cytokine pattern associated with anterior cruciate ligament (ACL) injury also suggests involvement of inflammatory cytokines in the onset and progression of PTOA [13, 14]. Of the various meniscal insults, bucket-handle (BH) meniscal tears are diagnosed in approximately 10–26% among all meniscus tears [15], whereas posterior-horn (PH) tears of the medial meniscus occur in approximately 28% of all meniscal tears [16, 17]. However, it is striking that PH is cited in almost 80% of patients with OA [18].

The goal of this study was to assess whether the expression of inflammatory and anti-inflammatory cytokines in the synovial fluid is influenced by the type of meniscal tear in medial meniscus injuries. PH tears are characterized by simpler tear patterns in terms of size and severity compared to BH tears.

We thought it was interesting to specifically study these two types of tears because the patients have different clinical presentations. Patients with BH tear usually present a more altered range of motion with respect to PH, usually presenting joint lock, without the possibility of fully extending the knee due to the displaced fragment. Consequently, we investigated the pattern of inflammatory and anti-inflammatory cytokine expression in the synovial fluid of patients who underwent knee arthroscopic surgery because of BH or PH tears of the medial meniscus. In particular, the synovial fluid concentrations of TNF-*α*, IL-1*β*, IL-6, IL-8, and those of the anti-inflammatory cytokine IL-10 were assessed. This information has increased our understanding of the roles these cytokines play in the cascade of events following a knee injury of the meniscus that may eventually lead to PTOA.

## 2. Methods

### 2.1. Subjects

Forty-five patients with medial meniscal tears, including 13 females and 32 males, ranging in age from 17 to 71 years old (mean = 52.2 years and median = 54 years) participated in this study. BMI was calculated for each patient. The principal requirement for inclusion was diagnosis of a medial meniscal tear (isolated or associated with cartilage damage). A BH lesion was defined as a vertical longitudinal tear of the meniscus with central displacement of the torn inner fragment. By contrast, a PH lesion was defined as including all posterior horn lesions, with the exception of any root lesions which were accompanied by a complete medial meniscus posterior root avulsion or a radial tear adjacent (within 9 mm) to the medial meniscus posterior root [19]. A BH tear was characterized by a larger tear size then PH tear. All diagnoses were made by a senior orthopedic surgeon based on (i) the history of present illness, (ii) a physical examination, (iii) magnetic resonance imaging (MRI), and, lastly, (iv) visual confirmation of the tear by arthroscopic examination. Chondral damage was evaluated arthroscopically and quantitated using the Outerbridge scoring system, where 0 = normal articular cartilage, I = superficial softening, II = superficial fissuring or fibrillation involving <1.5 cm area, III = fibrillation or fissuring involving >1.5 cm area, and IV = full-thickness cartilage wear with exposed subchondral damage [20]. Knees were graded based on the most severe area of chondral damage. As previously published, patients were divided in three groups on the base of the chondral damage: 0, I-II, and III-IV according to the Outerbridge scoring system [21].

Exclusion criteria included (i) a previous history of knee injury or infection, (ii) a preexisting inflammatory or arthritic disease(s), (iii) a systemic inflammation at the time of the collection of the sample, (iv) any anterior and/or posterior cruciate ligament or collateral ligament injury, (v) osteoarthritis, (vi) gout or pseudogout, or (vii) any previous intra-articular injection of any drugs. We measured time following injury on cytokine production, by resolving the patient population into two sample groups based on duration between injury and arthrocentesis; specifically, these were defined as (i) an acute phase, of 0 to 90 days, and (ii) a chronic phase, of greater than 90 days.

All patients provided written informed consent to the retention of biological material that would have otherwise been discarded. The experimental protocol was approved by the local Ethical Committee and conforms to the principles outlined in the WMA Declaration of Helsinki.

### 2.2. Samples

Synovial fluid was aseptically drawn from the knee without any lavage at the beginning of the arthroscopic surgery. Synovial samples were firstly collected in tubes containing EDTA, immediately centrifuged at 3000 × g to remove cellular debris, and the supernatant was stored at -80°C until assayed [13, 21–23]. The levels of cytokines TNF-*α*, IL-1*β*, IL-6, IL-8, and IL-10 were measured in the synovial fluid using specific sandwich enzyme-linked immunosorbent assay (ELISA) according to the manufacturer's instructions (TNF-*α*, IL-1*β*, and IL-10 were from R&D Systems, Minneapolis, MN; IL-6 and IL-8 were from eBioscience, San Diego, CA, U.S.A.).

### 2.3. Statistical Analysis

Statistical analysis was performed using MATLAB (R2017a; MathWorks Inc.). Normality of the data set was investigated using the Shapiro–Wilk Test, and all of them were found to be nonnormally distributed. Therefore, nonparametric statistic tests have been performed.

We set up a multivariate regression, including as regressors (i) age, (ii) BMI index, and (iii) sex, to investigate whether cytokine expression was influenced by any of these regressors.

The influence of time from trauma (i.e., acute or chronic), chondral damage (i.e., 0, I-II, and III-IV according to the Outerbridge scoring system), and type of lesion (i.e., BH, PH) on cytokine concentrations was assessed on the basis of the Mann-Whitney *U*-test when two groups were compared or the Kruskall-Wallis test when three groups were compared.

Correlations among biochemical markers were assessed for significance using the nonparametric Spearman rank correlation coefficient test. We considered only large correlations according to Cohen directions [24].

For all statistical tests, a value of *p* < 0.05 was considered to be statistically significant.

## 3. Results

### 3.1. Influence of Age, BMI, and Sex on Cytokine Expression in Medial Meniscal Tears

Multivariate regression was performed, but none of the regressors considered (age, BMI, and sex) reached the threshold for statistical significance (i.e., *p* < 0.05).

### 3.2. Effects of Time from Trauma, Chondral Damage, and Type of Lesion on Cytokine Concentrations in Medial Meniscal Tears

The demographic characteristics of the patient population enrolled in the study are summarized in Table 1.

The synovial fluid concentrations of TNF-*α*, IL-1*β*, IL-6, IL-8, and IL-10 were not significantly different between the “acute” and “chronic” injured groups (Table 2). Similarly, there were no significant differences in cytokine concentrations among groups with varied degrees of chondral damage (Table 2). By contrast, in the case of TNF-*α*, as well as IL-1*β*, there were significant differences in their synovial fluid concentrations between BH and PH groups (Table 2).

**Figure 1:**
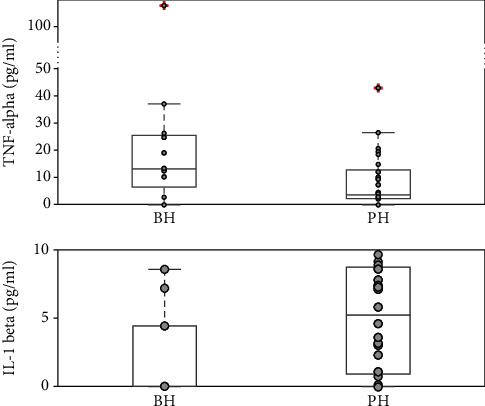
Modifications of cytokine levels in relation to the type of lesion. Cytokine concentrations measured in the population with medial meniscal tears (*n* = 45) that are significantly influenced by the type of lesion are represented in the box plots. In each plot, the box is built within the third (upper bound) and first (lower bound) quartiles (i.e., Q3, Q1); the middle line represents the median. Whiskers represent data maximum (upper whisker) and minimum (lower whisker). + indicates data outliers, defined as data points below Q1 − 1.5 × (Q3 − Q1) or above Q3 + 1.5 × (Q3 − Q1).

To illustrate, the synovial fluid concentration of TNF-*α* in the BH group was larger and significantly different (*p* = 0.031) from that in the PH group (Figure 1). By comparison, the synovial fluid concentrations of IL-1*β* in the PH group were larger and significantly different (*p* = 0.028) compared with those in the BH group (Figure 1). Levels of IL-6, IL-8, and IL-10 were not significantly different between the BH and PH groups (Table 3).

### 3.3. Correlations Between Cytokines in Knee Synovial Fluids

**Figure 2:**
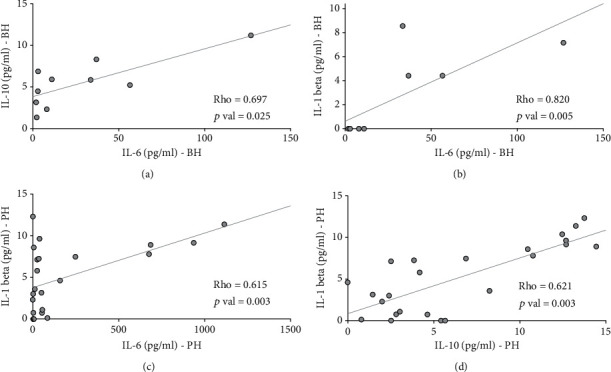
Correlation between cytokines in the synovial fluid of knees with medial meniscal tears. (a, b) Illustrate the significant correlations in the group of BH tears, whereas (c, d) illustrate the significant correlations in the group of PH tears. Correlations among the cytokines were assessed for significance using the nonparametric Spearman rank correlation coefficient test. A *p* < 0.05 was considered to be statistically significant. Calculated regression lines are shown.

We considered further the differential influence of BH and PH lesions on TNF-*α* and IL-1*β* levels by examining potential correlations between all the cytokines in the BH and in the PH groups. In the BH group (*n* = 12), there was a significant positive correlation between concentrations of IL-6 with IL-1*β* (rho = 0.802; *p* = 0.005) and those of IL-10 (rho = 0.697; *p* = 0.025) (Figure 2). In the PH group (*n* = 33), the mean levels of IL-1*β* were also positively and significantly correlated with the levels of IL-6 (rho = 0.615; *p* = 0.003) as well as with those of IL-10 (rho = 0.621; *p* = 0.003) (Figure 2).

## 4. Discussion

Since the number of possible variables along with the numerosity of our sample did not allow for a robust regression with all variables at a glance, a multivariate analysis with some parameters, such as age, sex, and BMI, that could possibly affect cytokine concentration was performed. The multivariate regression did not show any influence of these regressors on the cytokine levels. We therefore concluded that there was no confounding effect due to the aforementioned variables. In addition, given the multiple independent comparisons that did not show any effect on cytokine concentration, with the exception of the tear type, we can conclude that among all variables, only the tear type has an influence on cytokine concentrations.

In our study, we found that two of the most common types of meniscal lesions, BH and PH, result in two different inflammatory responses by the joint tissue. Synovial fluid concentrations of TNF-*α* and IL-1*β* levels, consequent to meniscal injury, were significantly different in the BH and PH groups. TNF-*α* and IL-1*β* are well-known proinflammatory cytokines which are involved in the pathogenesis of OA. TNF-*α* and IL-1*β* both activate intracellular signaling pathways that increase inflammatory (via NF-*κ*B) and catabolic processes in joint tissues [25]. At the cellular level, TNF-*α* and IL-1*β* reduce the efficiency of the respiratory chain and induce the production of iNOS, COX-2, and PGE2 synthase [26, 27]. TNF-*α* inhibits the synthesis of proteoglycans by chondrocytes [28] and stimulates the synthesis of metalloproteinases MMP-1, MMP-3, and MMP-13, which destroy cartilage, and ADAMTS-4, responsible for the proteolysis of aggregate molecules.

IL-1*β* stimulates the secretion of other cytokines such as TNF-*α*, IL-6, and IL-8 [29, 30]. The effects of IL-1*β* mainly affect cell metabolism and the extracellular matrix (ECM) [31, 32]. During the developing of OA, IL-1*β* stimulates the production of free oxygen radicals (ROS), which generate peroxides and hydroxylated radicals, that can directly damage the articular cartilage.

Meniscal tears have been proposed as the main precursor for the development of OA since tears of the posterior horn of the medial meniscus have been reported in about 80% of patients with OA [18]. Elevated levels of TNF-*α*, IL-1*β*, IL-6, IL-8, and IL-10 in the synovial fluid of knees with meniscal tears compared to healthy controls have been demonstrated previously [21, 33]. We decided to focus on the cytokine patterns expressed in two common but different meniscal lesions, such as BH and PH tears. BH tears are large lesions that cause mechanical locking when the central part of the tear is dislocated into the intercondylar notch. BH tears usually occur after trauma; also, PH tears may occur after a traumatic event, but sometimes, we can find a PH degenerative lesion caused by osteoarthritis with no trauma [34–37]. Considering the influence of OA in synovial cytokine levels, we included in the study only traumatic meniscal tears excluding patients with OA.

This study included only patients who presented with clinical symptoms that correlated with the MRI findings in their knee. We decided to consider only medial meniscal lesions and not lateral meniscal tears, because the latter differ in the traumatic mechanism and cause a different alteration of the joint biomechanics [38].

TNF-*α* is a proinflammatory cytokine of the acute phase of inflammation: in fact, the production of this cytokine is extremely rapid following the damaging event [39, 40]. It also plays a key role in regulating the inflammatory response: for example, it stimulates the production of adhesion molecules on the endothelial cells of the postcapillary venules (e.g., ICAM-1, VCAM-1, and E-selectin) [41]. Our results on TNF-*α* concentration levels in the BH group highlight its role following an acute traumatic event. In contrast, the expression of IL-1*β* is more complex and requires the transcription of pro-IL-1*β*, the activation of the inflammasome, and regulation by IL-1RA [42, 43]. This could be the reason for the higher levels of IL-1*β* measured in the PH group, since frequently, in this type of lesion, the meniscus is already partially degenerated before the trauma [44, 45], However, in a previous study [21], we did not find a relationship between the grade of chondral damage and cytokine levels.

The role of time elapsed from trauma was investigated in a longitudinal study of patients treated with reconstructive anterior cruciate ligament surgery. IL-6, IL-8, and IL-10 levels were high shortly after trauma and decreased in time, whereas synovial levels of TNF-*α* and IL-1*β* did not change with time [13]. However, one study reported that TNF-*α* was the only cytokine that remained significantly elevated at 5 year follow-up after the injury [46]. In the present study, there were 6 acute and 6 chronic samples in the BH group.

In a murine model of arthrosis following joint fracture, IL-1*β* was the main mediator of the acute phase of inflammation, particularly in the first 3 days [47]. A study examining the metabolic response of the meniscus to IL-1*β* in a canine model showed that the stimulation of the meniscal explants throughout the cytokine IL-1*β* compared to nonstimulated samples was brought to an important elevation of inflammatory biochemical mediators, such as NO, PGE2, IL-6, IL-8, MMP-3, and MMP-13; in addition, the same study showed also a significant reduction of the tissue GAG content [48]. However, another study on anterior cruciate ligament tears [22] showed that IL-1*β* levels were moderately higher in the acute phase than in healthy controls but then remained stable over time, suggesting a role of IL-1*β* in the inflammatory process not only in the acute phase but also in the chronic phase. Our findings obtained in the PH group are in agreement with these results.

We acknowledge the limitations of the present study. We are aware of the small size of the selected population: our attempt was to study only medial tears, resulting in reducing the number of subject enrolled in the study. For ethical reasons, we had no access to synovial samples from healthy knees, and so we choose to compare BH tears of medial meniscus with a group of patients with PH of medial meniscus. We also think it would have been appealing to study the correlation between the cytokine levels and clinical outcomes by using specific scores (i.e., Lysholm Knee Score) or else directly by studying the correlation between cytokine levels and clinical (i.e., pain, swelling, and instability) and functional aspects (i.e., limping, climbing and stairs) [49].

A multicentric study with a large population is recommended to confirm our results and to improve knowledge about the biochemical environment after BH tears.

Our results demonstrate that in the BH group, IL-6 was positively correlated with IL-1*β* (rho = 0.802; *p* < 0.01) and IL-10 (rho = 0.697; *p* < 0.05). This correlation is interesting since IL-6 is believed to be a primary proinflammatory cytokine which plays a key role in subchondral bone changes. IL-10 is an anti-inflammatory cytokine with chondroprotective effects that stimulates the synthesis of glycosaminoglycans, IL-1ra, and soluble TNF receptors but which also inhibits MMP-1, MMP-13, TNF-*α*, and IL-1*β* [50]. The positive correlation between IL-6 and IL-10 in the BH group suggests that a protective pathway is activated to antagonize the inflammatory biological response. Moreover, in the PH group, we found that IL-1*β* concentrations were positively correlated not only with IL-6 (rho = 0.615; *p* < 0.01) but also with IL-10 (rho = 0.621; *p* < 0.01). These correlations, between different pro- and anti-inflammatory cytokines, suggest the existence of a very complex regulation that needs more specific and targeted studies in order to be understood.

## 5. Conclusions

In conclusion, our data confirm the existence and relevance of the biochemical-inflammatory phenomenon that occurs after a meniscal tear. Patients with BH lesions have a cytokine profile in synovial fluid similar to that of patients with PH tears. However, significantly higher levels of TNF-*α* in BH tears and IL-1*β* in PH tears suggest a different role of the two cytokines in the two types of lesions. Further studies are needed to better understand the relationship between the biochemical inflammatory response subsequent to a meniscal injury and its possible role in determining OA alterations of the knee joint.

## Figures and Tables

**Table 1:** Characteristics of patients with bucket-handle (BH) tear and tear of the posterior horn (PH) of the medial meniscus.

		BH	PH	Total
Gender	M	12	20	32
	F	0	13	13
	Total	12	33	45

Age	Mean	40	56	
	Range	17-61	44-71	

BMI	Mean	22.6	23.7	
	Range	18-25	22-26	

Grade of chondral damage	0	8	6	14
	I-II	1	7	8
	III-IV	3	20	23

Time	Acute	6	2	8
	Chronic	6	31	37

**Table 2:** Effects of time from trauma, chondral damage, and type of lesion on the cytokine levels of medial meniscal tears.

Cytokine	Time from trauma (*p* value)	Chondral damage (*p* value)	Type of lesion (BH-PH) (*p* value)
IL-6	0.913	0.309	0.350
IL-8	0.860	0.789	0.988
TNF-*α*	0.364	0.398	0.031^∗^
IL-10	0.807	0.152	0.925
IL-1*β*	0.344	0.151	0.028^∗^

The influence of time from trauma and type of lesion on cytokine levels was assessed with the Mann-Whitney *U*-test, whereas the influence of chondral damage was assessed with the Kruskal-Wallis nonparametric test. The table shows the *p* value of the tests. ^∗^*p* < 0.05 for the BH vs. PH group.

**Table 3:** Cytokine concentrations.

		IL-6	IL-8	TNF-*α*^∗^	IL-10	IL-1*β*^∗^
BH	Median	22.3	41.2	13.2	5.8	0.0
	IQR	[3.0–74.0]	[7.9–159.4]	[8.3–24.9]	[3.8–7.5]	[0.0–4.4]
PH	Median	50.6	30.6	3.5	4.4	5.19
	IQR	[6.5–554.6]	[16.1–57.7]	[2.4–11.9]	[2.7–10.5]	[0.9–8.6]

Median and IQR (pg/ml) of synovial cytokines in patients with bucket-handle (BH) tear and tear of the posterior horn (PH) of the medial meniscus. ^∗^*p* < 0.05 for the BH vs. PH group.

## Data Availability

In order to access the data, please write to turati.mrc@gmail.com.
